# Residential flood vulnerability along the developed North Carolina, USA coast: High resolution social and physical data for decision support

**DOI:** 10.1016/j.dib.2019.103975

**Published:** 2019-05-22

**Authors:** Narcisa G. Pricope, Joanne N. Halls, Lauren M. Rosul, Christopher Hidalgo

**Affiliations:** University of North Carolina Wilmington, USA

## Abstract

This article presents an ArcGIS geodatabase of socio-demographic and physical characteristics derived from recent high resolution data sources to construct measures of population vulnerability to inundation in the 28 counties of coastal North Carolina, U.S.A. as presented in Pricope et al., 2019. The region is simultaneously densely populated, low-lying and exposed to recurrent inundation related to storms and incremental sea level rise. The data presented here can be used as a decision support tool in coastal planning, emergency management preparedness, designing adaptation strategies and developing strategies for coastal resilience. The socio-demographic data (population and housing) was derived from 228 tables at the block-group level of geography from the 2010 U.S. Census Bureau. These data were statistically analyzed, using Principal Component Analysis, to identify key factors and then used to construct a Social Vulnerability Index (SOVI) at the block-group level of geography which highlighted regions where socio-demographic characteristics such as family structure, race, housing (primarily owner vs. renter-occupied), special needs populations (e.g. elderly and group living), and household/family size play an overwhelmingly important role in determining community vulnerability from a social perspective. An index of physical exposure was developed using the National Flood Hazards Maps (available from North Carolina's Flood Risk Information System and FEMA) along with a novel building inventory dataset available from the North Carolina Department of Public Safety that contains the Finished-Floor Elevation of every structure in the state. We took advantage of the unprecedented high spatial resolution nature of the building inventory dataset to calculate an index of physical vulnerability to inundation of every block group in the 28 coastal counties relative to Base Flood elevations and identified hotspots where this intersection predisposes people to an increased risk of flooding. Here, we present the final derived dataset containing the social, physical and an integrative measure of vulnerability to flooding that can be used at multiple scales of analysis, starting with the regional, county, local, and neighborhood to identify areas of priority intervention for risk-reduction in coastal planning and emergency management preparedness as well as forward-looking adaptation strategies.

**Table:** Specifications table

Subject area	Geography and geospatial sciences; regional planning
More specific subject area	Geospatial assessments of population vulnerability to flooding
Type of data	Figures and database
How data was acquired	All data used in the analysis and presented here were derived from secondary datasets
Data format	Esri Vector Geodatabase
Experimental factors	Variables were normalized
Experimental features	Socio-economic data was reduced from over 3000 variables to 1000 and then subsequently further reduced through a principal components analysis
Data source location	Twenty-eight counties in coastal North Carolina, USA that makeup four regional management bodies (Councils of Governments); see Fig. 1
Data accessibility	The data is shared in this article and will also be made available on our university hosted server via FTP or an ArcGIS online platform
Related research article	Pricope, N.G., Halls, J. and Rosul, L. 2019. Modeling residential coastal flood vulnerability using finished-floor elevation and socio-economic characteristics. Journal of Environmental Management (in press) [1].

**Table:** 

**Value of the data** • The data presented here is a very first of its kind given it leverages a new and unprecedentedly high-resolution dataset of finished floor elevations for every building in North Carolina to create a highly resolved integrated model of social and physical vulnerability to flooding (coastal and pluvial) for North Carolina's densely populated coast. • The data presented here offers other researchers and planners the ability to conduct risk and vulnerability analyses, as well as engage in planning exercises that account for socio-demographic characteristics at the highest level of geography (block-group) while simultaneously being able to visualize the intersection of FEMA flood zones with finished-floor elevations of every building contained within the 100-year floodplain in 28 counties in North Carolina. • The methodology used to derived these data is transferable to other locations and regions with access to census and floodplain management datasets and, we hope, can open the door for collaborations with other stakeholders and researchers regionally and beyond. • The data can be combined with more in-depth survey data to understand people's perceptions of risk, to map public health concerns, or to gain a more nuanced understanding of under-served locations.

## 1 Data

**Fig. 1:**
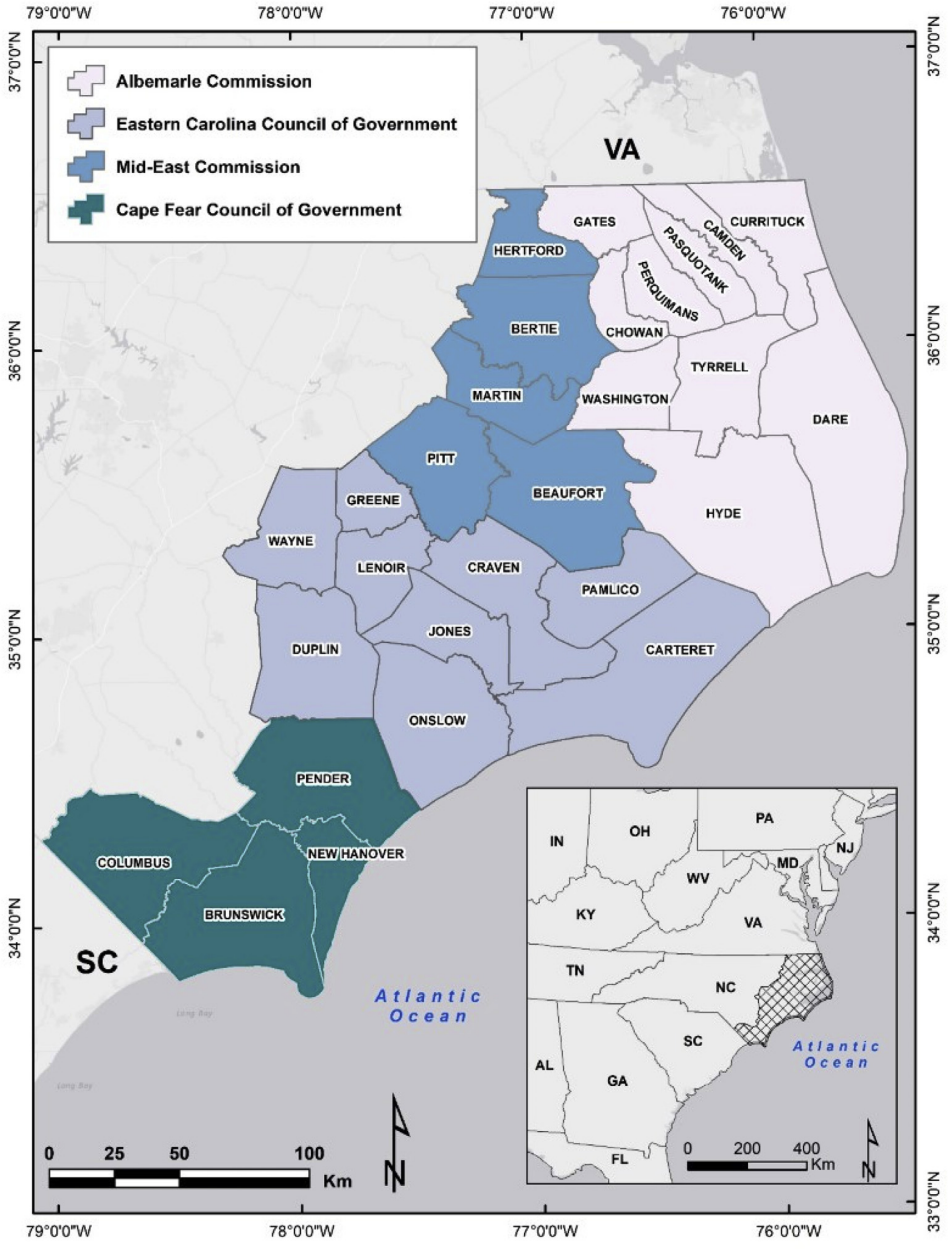
Regional Governments, counties, and study area within eastern North Carolina.

**Fig. 2:**
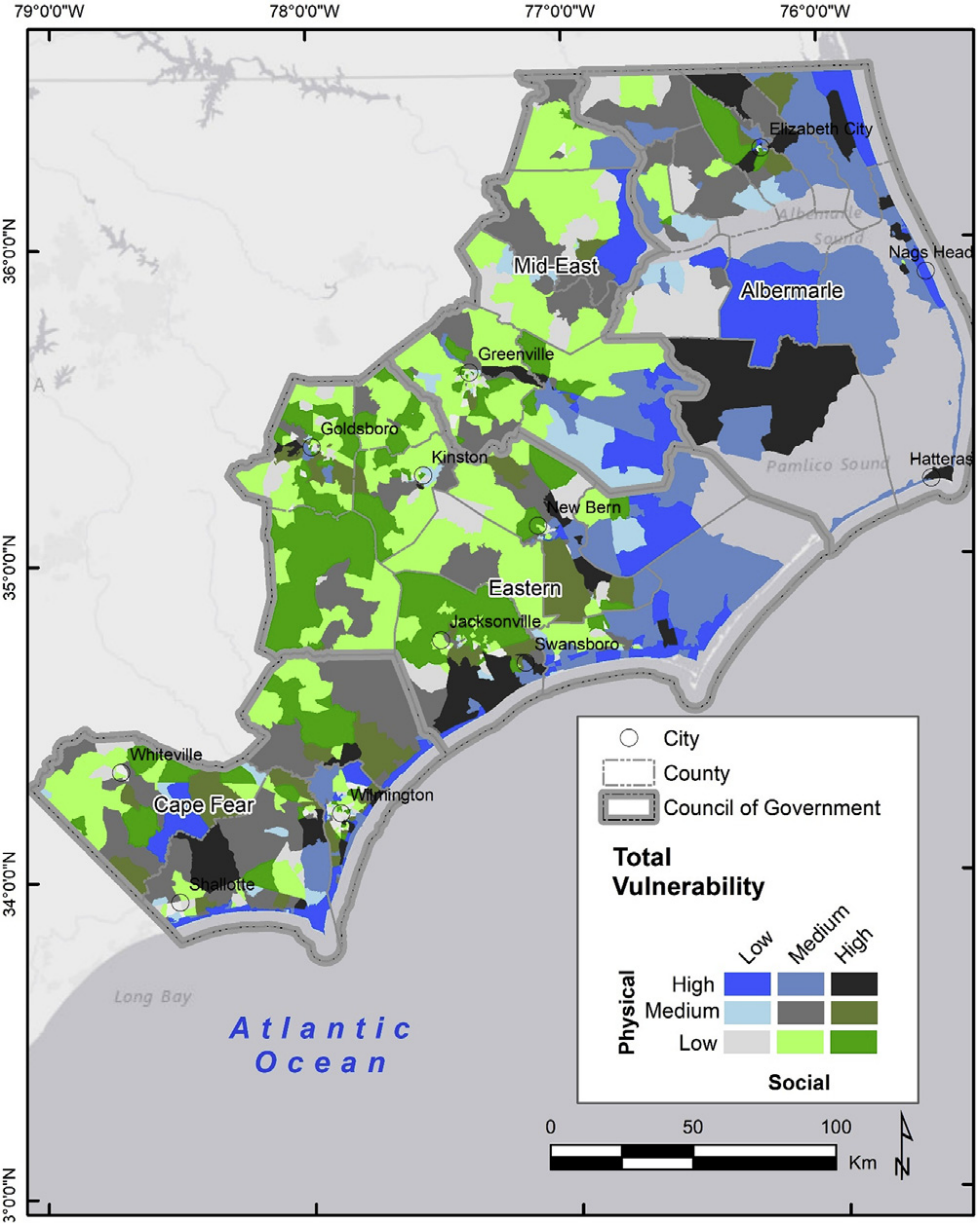
Spatial distribution of block-groups with high physical vulnerability and high weighted social vulnerability index values.

The data contains vector feature classes for each component of the project as referenced in Fig. 1 below, including a social, physical and combined, integrative index of vulnerability to inundation for 28 counties in North Carolina, USA (Table 1). The socio-demographic data was derived from 228 tables at the block-group level of geography from the 2010 U.S. Census Bureau and then used to create a social vulnerability index (SOVI). The physical exposure dataset relies on the National Flood Hazards Maps downloaded from North Carolina Flood Risk Information System along with a novel building inventory dataset available from the North Carolina Department of Public Safety that contains the finished-floor elevation of every structure in the state. Third, we present an integrated vulnerability index classified categorically to show areas of high, medium and low vulnerability (Fig. 2). Finally, we included the results of a clustering algorithm that tests the statistical significance of the integrative vulnerability spatial distribution (Table 2).

(Source: Pricope et al., 2019).

**Table 1:** Dataset, source, and data pre-processing.

Dataset	Source	Data pre-processing
2010 Census Summary File 1	US Census Bureau (American Factfinder) https://factfinder.census.gov	Data tables corresponding to the block-group GEOID's.
2010 TIGER/Line Shapefiles	US Census Bureau (TIGER/Line Shapefiles) https://www.census.gov/geo/maps-data/data/tiger-line.html	Subset the statewide block-groups to the study area.
Building Inventory	North Carolina Department of Public Safety	Subset the statewide dataset of 5,223,879 structure polygons to the study area.
Flood Hazard Maps	North Carolina Flood Risk Information System http://fris.nc.gov/fris/	Download and merge the 28 counties in the study area. The 100-year flood zones were selected and exported to a new feature class.

(Source: Pricope et al., 2019).

**Table 2:** Geodatabase (NCFloodRisk.gdb is located in Supplementary Materials) contains vector feature classes for each component of the project as referenced in the figures.

Feature class	Description	Fields
Albemarle	County polygons within the Albemarle Commission	County_Name
CapeFear	County polygons within the Cape Fear Council of Governments	County_Name
Eastern	County polygons within the Eastern Carolina Council of Governments	County_Name
Mideast	County polygons within the Mid-East Commission	County_Name
COGs	Council of Government boundaries	COG (numbered 1 through 4) Name (place name)
StudyArea	Polygon used for defining the study area extent	None
TotalPopBG	Total Population from 2010 United States Census by Block Groups in study area	GEOID (links to Census data) Total_Pop (population)
PCA_SOVI	12 Principal Component Analysis factors, by Block Group, and the computed SoVI scores.	GEOID, Factor1, Factor2, Factor3, Factor4, Factor5, Factor6, Factor7, Factor8, Factor9, Factor10, Factor11, Factor12, SOVI
SOVI_Clusters	Anselin Local Moran's I cluster attributes by Block Group	SOVI score, LMiIndex, LMiZscore, LMiPValue, COType, NNeighbors (See esri.com for detailed description of each field)
Physical	Physical vulnerability for the structures (FFE-BFE) and FEMA floodplains	PctStructure (percentage of structures that are vulnerable), PctFloodplain (percentage of BG with 100-yr floodplain), PVI (Physical Vulnerability Index), PVI_ZScore (PVI converted to Z Score)
Vulnerability	Combined SOVI and PVI by category (H = High, M = Medium, and L = Low)	PVI_Cat, SOVI_Cat, PVI_SOVI_Cat
Vulnerability_Cluster	Anselin Local Moran's I cluster analysis of total vulnerability (SOVI & PVI) by Block Group	TotalScore, LMiIndex, LMiZscore, LMiPValue, COType, NNeighbors (See esri.com for detailed description of each field)

## 2 Experimental design, materials, and methods

The experimental design, materials and methods are described in great detail in our paper (Pricope et al., 2019) and we invite readers to refer to that for the detailed methodology.
