# Supplementary material for: Residential flood vulnerability along the developed North Carolina, USA coast: High resolution social and physical data for decision support
Source: Data Brief. 2019 May 22;24:103975. doi: 10.1016/j.dib.2019.103975 (PMC6545379; doi:10.1016/j.dib.2019.103975)
Supplement: Multimedia component 1 [file mmc1.doc]

Conflict of Interest and Authorship Conformation Form

Please check the following as appropriate:

- X All authors have participated in (a) conception and design, or analysis and interpretation of the data; (b) drafting the article or revising it critically for important intellectual content; and (c) approval of the final version.
- X This manuscript has not been submitted to, nor is under review at, another journal or other publishing venue.
- X The authors have no affiliation with any organization with a direct or indirect financial interest in the subject matter discussed in the manuscript
- X The following authors have affiliations with organizations with direct or indirect financial interest in the subject matter discussed in the manuscript:

Author’s name Affiliation

Narcisa Pricope University of North Carolina Wilmington
